# The Impact of Goal Disturbance after Cancer on Cortisol Levels over Time and the Moderating Role of COMT

**DOI:** 10.1371/journal.pone.0135708

**Published:** 2015-08-27

**Authors:** Moniek Janse, Martijn van Faassen, Ido Kema, Ans Smink, Adelita V. Ranchor, Joke Fleer, Mirjam A. G. Sprangers

**Affiliations:** 1 Department of Health Psychology, University of Groningen, University Medical Center Groningen, Groningen, The Netherlands; 2 Department of Laboratory Medicine, University of Groningen, University Medical Center Groningen, Groningen, The Netherlands; 3 Department of Medical Psychology, University of Amsterdam, Academic Medical Center Amsterdam, Amsterdam, The Netherlands; Harbin Medical University, CHINA

## Abstract

Due to physical hindrance and time spent in hospital, a cancer diagnosis can lead to disturbance of personally important goals. Goal disturbance in cancer patients has been related to poorer psychological well-being. However, the relation with physiological measures is yet unknown. The purpose of the current study is to examine the impact of goal disturbance on cortisol as a measure of response to stress over time, and a possibly moderating role of a DNA genotype associated with HPA-axis functioning, Catechol-*O*-Methyl transferase (COMT). We examined the predictive value of goal disturbance on Cortisol Awakening Response (CAR) and Diurnal Cortisol Slope (DCS) over two periods: 1–7 and 7–18 months post-diagnosis, and the moderating role of COMT during these periods. Hierarchical regression analyses showed that goal disturbance 7 months post-diagnosis significantly predicted a steeper CAR a year later. During that period, the slow COMT variant moderated the relation, in that patients reporting high goal disturbance and had the Met/Met variant, had a more flattened CAR. No other significant effects were found. As steeper CARs have been related to adverse health outcomes, and COMT genotype may modify this risk, these results indicate that goal disturbance and genotype may be important factors to consider in maintaining better psychological and physical health in the already vulnerable population of cancer patients.

## Introduction

The successful pursuit of personal goals is important for maintaining well-being [1], as goals provide a sense of purpose in life and motivate daily activities [2,3]. The diagnosis of a potentially life-threatening disease as cancer may lead to goals being more difficult to achieve due to the physical implications of the illness and/or its treatment [4,5]. Evidence suggests that this disturbance of personal goals leads to decreased levels of psychological well-being in cancer patients [4,6,7].

A less researched question concerns the relationship between goal disturbance after cancer and physiological measures. In one study in healthy people, it was found that hindrances in goal attainment led to increased levels of cortisol [8], providing a first indication that goal disturbance can be considered a stressor impacting physiological outcomes. Cortisol is a hormone which secretion is regulated by the hypothalamo-pituitary-adrenal (HPA) axis and can be used to measure the response to stress. The HPA-axis is a pathway through which psychological factors can influence biological processes [9], and stress has been found to influence cortisol production [10,11]. Indeed, worries and daily stressors, like goal disturbance after cancer diagnosis, have been linked to steeper Cortisol Awakening Responses (CAR, increase in cortisol between awakening and 30–45 minutes later) [12–17] and flattened Diurnal Cortisol Slopes (DCS, decrease in cortisol between 30–45 minutes after awakening and bedtime) [18–22], which have been related to lower levels of psychological as well as psychical well-being [20,23–26]. In the current study, we will investigate the longitudinal impact of goal disturbance due to cancer on cortisol levels.

Catechol-*O*-Methyl transferase (COMT, EC 2.1.1.6) is an enzyme involved in the degradation of catecholamines. It is encoded by the COMT gene (chromosome 22 band q11.2) that displays several allelic variants of which the functional single nucleotide polymorphismVal^158^Met is most studied [27,28]. This functional polymorphism in the COMT gene is the result of a G to A mutation that translates into a valine (val) to methionine (met) substitution at codon 158. It has been shown to account for a four-fold decrease in enzyme activity in degrading cortical dopamine (catecholamines) [29]. Catecholamines modulate the endocrine stress reactivity through its impact on those parts of the brain that regulate the functioning of the HPA-axis [30]. Therefore, the genetic differences in the dopaminergic system (as modified by e.g. COMT) can lead to differences in the reactivity of the HPA-axis. As the HPA-axis regulates cortisol secretion, the COMT variants may have influence on cortisol reactivity [31,32]. In the general population, three common variants, Val/Val, Val/Met, and Met/Met, have been identified [33]. The low-activity Met/Met genotype has been associated with reduced COMT enzyme activity in the brain [29,32]. Low enzyme activity leads to an impaired endocrine response to stress [28]. It could thus be that, when experiencing stress, Met/Met carriers would show a more flattened CAR and DCS, as they are less able to degrade catecholamines, leading to lower HPA-axis reactivity and an impaired ability to regulate or degrade cortisol levels. Therefore, it could be hypothesised that cancer patients who experience stress through goal disturbance and have the Met/Met genotype, would show more flattened CAR and DCS than cancer patients with a Val/Val or Val/Met genotype. In the present study, we investigated to what extent the COMT Val^158^Met polymorphism moderates the relation between goal disturbance and cortisol levels.

The present study is the first to undertake a longitudinal analysis of goal disturbance and cortisol levels in cancer patients, and the moderating role of COMT. We hypothesise that 1) higher goal disturbance within a month after diagnosis predicts a steeper CAR and flattened DCS six months later, and that higher goal disturbance seven months post-diagnosis predicts a steeper CAR and flattened DCS one year later. Additionally, we expect that 2) COMT genotype moderates the relation between goal disturbance and cortisol levels, in that the Met/Met variant would lead to a more flattened CAR and DCS after goal disturbance than Val/Val or Val/Met genotypes.

## Methods

### Sample

Recently diagnosed colorectal cancer patients were recruited between September 2011 and March 2013 in four different hospitals across the Netherlands. Patients had to be over 18 years old to be eligible. Those who had a cognitive impairment or psychiatric disorder, were unable to understand Dutch, or had drug or alcohol problems, were excluded from participation. The study included three assessments: within a month, 7 and 18 months post-diagnosis. At all three assessments, patients were interviewed by the same interviewer at a place of their choice (usually their homes). The study was approved by the Medical Ethical Committee of the University Medical Center in Groningen, the Netherlands, and all participants provided written informed consent. This consent procedure was approved by the ethical committee.

### Measures

#### Demographic and clinical variables

At the first assessment, patients reported their age, gender, marital status and highest level of educational attainment. From the Netherlands Cancer Registry (NCR), we obtained information on prognosis (using the Tumour Node Metastasis (TNM) Classification of Malignant Tumours, ranging from Stage I, local tumour without metastases, to Stage IV, invasive tumour with metastases), presence of a stoma (yes/no), site of the tumour (colon or rectum), and whether patients underwent chemo—and/or radiotherapy in addition to surgery during the course of the study (yes/no).

#### Goal disturbance

In a recent review on life goals in cancer patients, goal disturbance was identified as the most studied aspect of the goal construct [34]. However, the review also showed that there is no ‘golden standard’ or validated scale that was used most often. A common way to assess dimensions or structural aspects of personal goals, such as goal disturbance, is to first assess personal goals, and then let respondents appraise these goals on structural aspects (e.g. goal importance, effort, or disturbance) [2,3,35]. Indeed, most studies assess goal disturbance by asking respondents to rate their goals (either freely elicited or from a pre-defined list of goals) on goal disturbance or goal hindrance (e.g. [4,36–39]). The present study therefore assesses goal disturbance by first asking patients to freely list three to ten personal goals, and then rate for each goal the extent to which cancer hindered the achievement of that goal on a 10-point scale (ranging from 1 = not at all, 10 = completely). Mean goal disturbance scores from all goals were then calculated for each assessment.

#### COMT genotype and cortisol

During the first assessment (i.e. within one month post-diagnosis), COMT genotype was assessed in salivary DNA samples obtained with DNA Collection kits (Oragene by Genotek). The interviewer took a kit to the interview and the patient was asked to fill the cup with saliva. The interviewer then mailed the cup to the researchers at the University Medical Center Groningen, who took it to the Department of Laboratory Medicine. Mailing of the DNA samples is not known to affect their quality [40].

Seven and 18 months post-diagnosis, saliva was collected to assess cortisol. Collection kits (containing salivettes, see below) were mailed to the participants prior to these assessments. Patients collected three samples per day for two consecutive days at both assessments: immediately upon waking, 30 minutes later, and at bedtime. All samples were numbered consecutively by time and day by the researchers before they were sent. Patients were telephoned by a member of the research team to inform them that they would receive a saliva collection kit with a detailed instruction sheet. During the call, patients were instructed how to collect the samples and asked to do so in the week before the interviewer would come to visit them. Patients were reminded of the importance of compliance with the instructions and told to store the collected samples in the refrigerator. After the interviewers had conducted the interviews, they collected the samples and mailed the collection kits to the Department of Laboratory Medicine at the University Medical Center Groningen. Those patients who did not complete saliva collection prior to the interview, mailed the kit themselves in a pre-paid envelope. Mailing of samples to the laboratory has not been known to affect the quality of the samples [41], and this procedure is commonly used in similar studies [9].

Patients had to place a synthetic cotton roll in their mouth (Sarstedt Salivettes), chew until it was saturated with saliva and return the cotton in a vial. They were asked to collect the samples on weekdays (due to the differences in CAR between weekdays and weekends [13]), and not to brush their teeth or eat within 30 minutes before collection to avoid contamination of the sample with blood or food. Patients were instructed to write down the exact time of collection of each saliva sample, as well as the exact times of awakening and bedtime, on a registration form provided by the researchers. They were also asked to indicate whether they had diabetes or not, as this may influence cortisol levels [42]. Cortisol was analysed by isotope dilution mass spectrometry, essentially as described in Jones et al. [43]. Intra-assay CV were 8.6%, 2.1%, and 2.1%, inter-assay CV were 9.6%, 5.2%, and 4.5% at respectively 0.48, 2.7, and 11 nmol/L.

### Data analysis

Cortisol values were not normally distributed, and therefore we used a logarithmic transformation to normalise the data [9]. CAR was examined by calculating the difference between the sample collected immediately upon awakening and 30–45 minutes later [16,44–46]. To increase the reliability and reduce situational influences, CAR scores of the two consecutive days were averaged to arrive at one CAR per assessment [47]. We calculated the DCS by subtracting the bedtime sample from the sample collected 30–45 minutes after awakening and divided this by the number of hours passed [9,16,44]. The DCS of the two consecutive days were averaged to form the DCS per assessment.

A decrease in CAR, and therefore a negative CAR value, could occur either due to a natural reaction, or due to non-adherence with respect to time of sample collection. To control for possible non-adherence or other unknown variables which can affect CAR, we followed Almela et al.’s approach (based on [48]) by replicating analyses for all patients who showed a positive CAR (and therefore the expected increase in cortisol) on both days per assessments (the 2-day CAR group), and those who did so at only one or none of the days (the 1–0 or 0–0 day CAR group).

Several factors have been suggested to influence cortisol levels [49,50], and should therefore be taken into account. Among the most commonly studied factors are age and gender, participant adherence, weekdays vs. weekend collections, sleep duration, and time of waking. Participant adherence, weekdays vs. weekend collections, sleep duration and time of waking were controlled for when preparing the sample. Age and gender were controlled for in the analyses. In addition, HPA-axis functioning can be affected by psychiatric disorders [51]. In the current study however, presence of a psychiatric disorder was an exclusion criteria. This factor will therefore not influence the study’s findings.

First, we used descriptive statistics to investigate the characteristics of the sample. Hierarchical regression analyses were used to assess the predictive value of goal disturbance on CAR and DCS from within one month to 7 months post-diagnosis, and from 7 months post-diagnosis to 18 months post-diagnosis. Since our primary interest was the relation between goal disturbance and cortisol levels, we tested their correlations with demographic and clinical variables, to investigate whether we should control for any of these variables in the regression analyses. No significant correlations were found between cortisol levels, age, gender, stage of cancer, and whether they received chemo- and or radiotherapy during the course of the study. However, as age and gender have been suggested as factors that could influence cortisol levels, these factors were entered in step one of the analyses. Goal disturbance was entered in step two. As COMT is a categorical variable with three categories, it was recoded using two dummy variables (Val/Val 0 = no, 1 = yes and Met/Met 0 = no, 1 = yes, with the intermediate active Val/Met type as reference value) and entered in step three of the regression analyses [52]. To investigate and interpret a possible interaction effect, we first centered the predictor goal disturbance by subtracting the mean from each data point, so that the new centered variables have zero as the mean. We then calculated the two interaction variables using the two dummy variables and entered these in step four of the regression analysis. We used an alpha of 0.05 to test for significance (2-sided). Statistical Package for Social Sciences (IBM SPSS) version 22.0 for Windows was used for the statistical analysis.

## Results

### Sample characteristics

In total, 497 patients were offered information concerning the study. Of these patients, 117 refused this information. Of the 380 patients who received the information, 228 agreed to participate (response rate: 45.9%), 219 completed the first assessment, and 186 patients completed all three assessments (drop-out: 15.1%). All patients were asked to collect saliva, for DNA isolation for COMT analysis and for cortisol analysis, but of the 186 patients who completed all assessments, ten patients did not provide consent for COMT, cortisol, or both, at the start of the study. Eighteen patients were unable to collect cortisol 7 months post-diagnosis and fifteen 18 months post-diagnosis, mostly due to the severity of their illness. Of the 143 patients who collected cortisol, 114 returned more than 50% of their samples, which were of sufficient quality to be analysed. Fifteen patients were then excluded because the time registration was filled out incompletely (less than 50%) or was not returned with the samples, and one further patient was excluded as no cortisol was present in the returned samples, leaving suitable samples of 98 patients.

**Fig 1:**
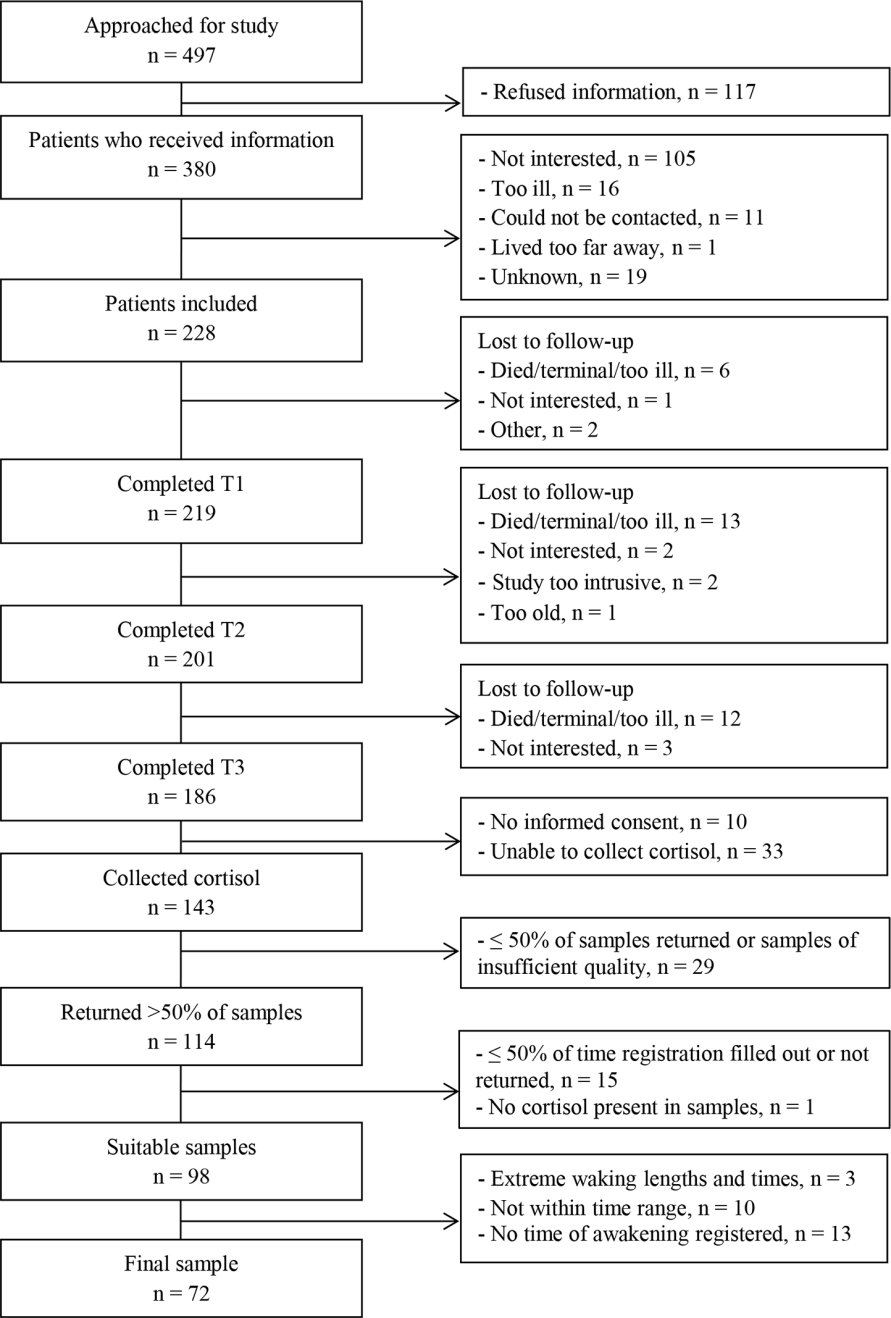
Flowchart of patients who provided useful samples.

Following Karlamangla et al. [53], we then excluded respondents with extreme waking day lengths (> 20h) (n = 1) and extreme waking times (before 04:00 AM and after 11:00 AM) (n = 2). Additionally, given the importance of time elapsed since awakening on cortisol awakening response [54], we excluded participants who, at any day, failed to collect the first saliva sample within 30 minutes after awakening and the second saliva sample within 30 minutes after the first assessment with a margin of plus or minus 15 minutes (n = 10). Patients who failed to register time of awakening were also excluded because we could not be sure whether the saliva sample was collected within 30–45 minutes after awakening (n = 13). We therefore could analyse cortisol samples of 72 patients (see Fig 1 for the complete flowchart).

Table 1 presents the demographic characteristics. The 72 patients were, on average, 65.2 years old, mostly male (58.3%), with a partner (83.3%), and completed at least high school (63.9%). Most patients were diagnosed with either Stage II or III colorectal cancer, did not have a stoma and had a tumour located in the colon. Almost half of patients (47.2%) received chemotherapy or radiotherapy in addition to surgery during the study. For two patients, COMT genotype could not be determined. Of the remaining patients, 16 (22.2%) had the Val/Val genotype, 32 (44.4%) the Val/Met genotype and 22 (30.6%) the Met/Met genotype. Three patients reported to have diabetes. The demographic characteristics did not differ significantly from the original sample of 186 patients, except with respect to presence of a stoma: patients in the current sample less often had a stoma compared to those in the original sample. Age, gender and stage of illness also did not differ from the 219 patients who completed the first assessment.

**Table 1:** Patients’ demographics.

Variable	No. (%)
**Age, yrs, mean (SD)**	65.2 (10.7)
**Female**	30 (41.7)
**With partner**	60 (83.3)
**Education**	
***Low (primary school/LBO)***	11 (15.3)
***Medium (high school/MBO)***	35 (48.6)
***High (college/university)***	26 (36.1)
**Prognosis (TNM stage)**	
***Stage I***	17 (23.6)
***Stage II***	24 (32.3)
***Stage III***	24 (32.3)
***Stage IV***	5 (6.9)
**Presence of stoma (yes)**	16 (22.2)
**Site of tumour (colon)**	45 (62.5)
**Chemo- and/or radiotherapy (yes)**	34 (47.2)
**COMT**	
***Val/Val***	16 (22.2)
***Val/Met***	32 (44.4)
***Met/Met***	22 (30.6)

(n = 72) (percentages may not add up to 100% due to missing data).

Table 2 presents data on goal disturbance, mean levels of cortisol, and CAR and DCS over time. Scores on goal disturbance ranged from 1–10. The sample of 72 patients did not differ significantly on levels of goal disturbance from the 114 patients who dropped out or were excluded at all three assessments. At the second assessment, 40 patients (54.1%) showed a positive CAR on both days, while at the third assessment, 30 patients (40.5%) showed a positive CAR on both days. For DCS, 3 samples were excluded as they showed an increase of more than 10 nmol/L, as this may reflect that the second sample was not captured at the cortisol peak [44]. There were no significant differences in CAR and DCS over time. At both assessments, there were no significant COMT group differences in mean levels of cortisol and CAR. The only significant group differences however, were with respect to the DCS at the second assessment, where patients with the Met/Met variant showed the most flattened DCS compared to the other two variants (*p* <.001).

**Table 2:** Data for goal disturbance, cortisol levels, CAR, DCS, and COMT over time (n = 72). Paired samples t-tests were used to test significant differences between time 2 and time 3, and ANOVA’s with COMT genotype as between-group factor to test significant differences in cortisol levels between genotypes.

Variable	M (SD) Time 1:	M (SD) Time 2:	M (SD) Time 3:	t (*p*)
	Within 1 month post- diagnosis	7 months post-diagnosis	18 months post-diagnosis	
**Goal disturbance**	4.9 (2.5)	4.0 (2.7)	3.3 (2.5)	
**Cortisol samples (nmol/L)**		9.9 (5.6)	9.1 (4.3)	1.51 (.13)
Val/Val		10.95 (6.45)	9.58 (3.07)	
Val/Met		10.12 (6.41)	9.15 (5.59)	
Met/Met		8.81 (3.39)	8.81 (3.26)	
** F (*p*)**		.71 (.5)	.13 (.88)	
**CAR**		.38 (.53)	.28 (.61)	1.23 (.22)
Val/Val		.25 (.32)	.09 (.48)	
Val/Met		.45 (.53)	.30 (.70)	
Met/Met		.32 (.64)	.35 (.53)	
** F (*p*)**		.90 (.41)	1.0 (.37)	
**DCS**		-.16 (.06)	-.15 (.05)	-1.34 (.18)
Val/Val		-.17 (.5)	-.14 (.05)	
Val/Met		-.18 (.06)	-.15 (.06)	
Met/Met		-.12 (.06)	-.15 (.04)	
**F (*p*)**		**7.93 (<.001)**	.33 (.72)	

### Prediction of goal disturbance on cortisol levels (hypothesis 1)

Tables 3 and 4 present the results of the hierarchical regression analyses on the predictive value of goal disturbance on cortisol at the second and third assessment, respectively. Goal disturbance within a month following cancer diagnosis was insignificantly related to either CAR or DCS six months later (see Table 3). Using only the group who showed an increase in CAR on both days of the assessment (the 2-day CAR group) to predict CAR 7 months post-diagnosis did not show any significant results (results not shown). In line with our hypothesis, a high level of goal disturbance seven months post-diagnosis was significantly related to steeper CAR one year later (see Table 4). Using only the 2-day CAR group to predict CAR 18 months post-diagnosis did not show any significant results (results not shown).

**Table 3:** Hierarchical regression analyses predicting cortisol 7 months post-diagnosis (Time 2).

	Step 1		Step 2		Step 3		Step 4	
	B (SE)	Beta	B (SE)	Beta	B (Se)	Beta	B (Se)	Beta
**CAR Time 2**								
Age	-.01 (.01)	-.03	.00 (.01)	.01	.00 (.01)	.02	.00 (.01)	.02
Gender	-.02 (.13)	-.02	-.04 (.13)	-.04	-.07 (.13)	-.07	-.06 (.14)	-.06
Goal disturbance Time 1	-		.03 (.03)	.13	.03 (.03)	.15	.03 (.04)	.15
Val/Val	-		-		-.24 (.17)	-.19	-.21 (.17)	-.17
Met/Met	-		-		-.13 (.15)	-.12	-.12 (.15)	-.11
Goal disturbance × Val/Val	-		-		-		-.03 (.08)	-.07
Goal disturbance × Met/Met	-		-		-		.02 (.06)	.04
ΔR^2^	.00		.02		.03		.01 (total = .05)	
**DCS Time 2**								
Age	.00 (.00)	.07	.00 (.00)	.10	.00 (.00)	.08	.00 (.00)	.06
Gender	.02 (.02)	.19	.02 (.02)	.18	.03 (.01)	.21	.03 (.02)	.22
Goal disturbance Time1	-		.00 (.00)	.10	.00 (.00)	.12	.00 (.00)	.03
Val/Val	-		-		.01 (.02)	.08	.01 (.02)	.08
Met/Met	-		-		.07 (.02)	**.49** **	.07 (.02)	**.50** **
Goal disturbance × Val/Val	-		-		-		.00 (.01)	.04
Goal disturbance × Met/Met	**-**		-		-		.01 (.01)	.11
ΔR^2^	.04		.01		**.21** **		.01 (total = .27)	

*p < 0.05

**p < 0.01

**Table 4:** Hierarchical regression analyses predicting cortisol 18 months post-diagnosis (Time 3).

	Step 1		Step 2		Step 3		Step 4	
	B (SE)	Beta	B (Se)	Beta	B (Se)	Beta	B(Se)	Beta
**CAR Time 3**								
Age	-.01(.01)	-.18	-.01 (.01)	-.18	-.01 (.01)	-.17	-.01 (.01)	-.17
Gender	.11 (.15)	.09	.11 (.15)	.09	.10 (.15)	.08	.06 (.14)	.05
Goal disturbance Time 2	-		.01 (.03)	.03	.01 (.03)	.02	.09 (.04)	**.39** *
Val/Val	-		-		-.19 (.19)	-.13	-.20 (.18)	-.14
Met/Met	-		-		.07 (.17)	.06	.06 (.16)	.05
Goal disturbance × Val/Val	-		-		-		-.08 (.07)	-.16
Goal disturbance × Met/Met	**-**		-		-		-.18 (.06)	**-.48** **
ΔR^2^	.04		.00		.03		.12 (total = .19)	
**DCS Time 3**								
Age	.00 (.00)	-.08	.00 (.00)	-.11	-.00 (.00)	-.11	.00 (.00)	-.11
Gender	.01 (.01)	.06	.01 (01)	.08	.01 (.01)	.10	.01 (.01)	.10
Goal disturbance Time2	-		-.00 (.00)	-.18	-.00 (.00)	-.18	-.00 (.00)	-.08
Val/Val	-		-		.01 (.02)	.10	.01 (.02)	.09
Met/Met	-		-		-.00 (.01)	-.03	-.00 (.02)	-.03
Goal disturbance × Val/Val	-		-		-		-.01 (.01)	-.13
Goal disturbance × Met/Met	**-**		-		-		-.00 (.01)	-.07
ΔR^2^	.01		.03		.01		.01 (total = .06)	

*p < 0.05

**p < 0.01

### Moderating role of COMT of the relation between goal disturbance and cortisol levels (hypothesis 2)

Interaction analyses were performed to investigate the moderating role of COMT between goal disturbance and cortisol levels (see Tables 3 and 4). The main effect of having the Val/Val genotype, as well as having the Met/Met genotype, on cortisol was tested. The Val/Met genotype was used as the reference category. No significant interactions were found during the period 1–7 months post-diagnosis for either CAR or DCS. During the period 7–18 months post-diagnosis, results showed a significant interaction effect as the Met/Met genotype moderated the relation between goal disturbance 7 months post-diagnosis and CAR one year later. Patients who scored high on goal disturbance and had the Met/Met genotype, had more flattened levels of CAR than patients who were high on goal disturbance and had the Val/Met genotype. No interaction effects were found in the relation between goal disturbance and DCS. No interaction analyses were performed using only the 2-day CAR group, as group sizes would be too small.

## Discussion

The aim of the current study was to investigate the relation between goal disturbance and cortisol levels over time, and the moderating role of COMT herein. No significant relations were found during the first six months after cancer diagnosis. During the year thereafter however, we found indications that higher goal disturbance seven months post-diagnosis predicted steeper or increased CAR the year thereafter. The direction of this finding was in line with our hypothesis. No significant results were found with respect to the DCS. Additionally, in line with our hypothesis, moderator analyses showed that patients who indicated to have a high level of goal disturbance 7 months post-diagnosis were found to have more flattened CAR one year later if they had the slow-activity COMT genotype Met/Met. No other significant interactions were found.

The findings of the present study are the first to suggest a possible influence of goal disturbance after cancer on a physiological outcome (i.e. cortisol as a measure of response to stress). Although studies that examined the effect of perceived stress on CAR found mixed results, the evidence for an association between an increased CAR and chronic stress is growing and becoming more robust [22,50]. The current results support the assumption that disturbance of personally important life goals due to cancer can be considered a chronic stressor which affects physiological aspects as the CAR. This is in line with the finding that the significant predictive value of goal disturbance on CAR was only found between 7–18 months post-diagnosis, and not during the first months after diagnosis. Only after several months of living with goal disturbance, the stress may become chronic and the physiological effects visible. Chronic stressors may require that people need to restructure their lives [11], and need to live with ongoing challenging circumstances [18,55]. During the follow-up period, cancer patients may need to do exactly that, and face daily goal disturbance caused by the illness. Anticipation of these daily demands is also suggested to lead to an increased CAR [50].

No significant relation was found with respect to the DCS. As it is suggested that DCS may reflect different processes than CAR [56,57], it could be that psychological stressors less affect DCS. Given the responsiveness of CAR to psychological aspects, this measure is recommended for studying the relation between psychological stressors, such as goal disturbance, and biological responses [49]. More research is needed to examine which stressors may impact DCS.

Little is known about the role of the COMT genotype in the relation between stressors and CAR and DCS. The present study found that, in line with our hypothesis, carrying the Met/Met genotype led to a more flattened CAR over time when patients experienced more goal disturbance. These results are the first to suggest a role of DNA genotype in CAR after goal disturbance in cancer patients. They indicate that Met/Met carriers, compared to the other COMT variant carriers, may indeed have a relative insensitivity to modulating the endocrine stress reactivity, and a reduced capability to degrade cortisol secretion [30]. Previous research suggests that Met/Met carriers show inflexibility with adapting to changing situations and respond more inefficiently to worries with respect to their cortisol levels [28,58,59]. It could be suggested that only well after diagnosis, when permanent life changes are visible, the effect of COMT genotype becomes visible. However, it should be further investigated why this effect was only present in CAR 18 months post-diagnosis. Alternatively, Met/Met carriers might be unable to exhibit the required physiological stress response due to their low reactivity. Therefore, they may deal with goal disturbance more poorly overall and are more stressed.

The relationship between chronic stressors and adverse health effects is well established [18], and cortisol may affect psychological [60] as well as physical well-being [20]. For cancer patients, it seems especially important to maintain normative cortisol levels, as increased cortisol levels have been related to a reduced immune function, which may promote tumor growth [11,23], and prolonged cortisol secretion may lead to an increased risk for mortality in cancer patients [11,23]. The relationship between CAR and health effects is less well established [49], but there is evidence linking an increased CAR to several negative psychological and physical health outcomes [50,61,62]. If increased CAR is indeed related to negative health outcomes, it is especially important that cancer patients maintain a normative CAR and that goal disturbance is reduced.

Overall, studies found that carrying the Met/Met variant poses a risk factor for negative emotional and physiological outcomes [28,31], and that Val/Val carriers are more resilient to stress [28,58,59]. However, it remains difficult to say whether the Met/Met variant is detrimental for health when investigating CAR. Although flattened CARs are overall related to better health outcomes, prolonged high cortisol levels may have adverse implications [11,23]. However, we did not find that patients in our sample with the Met/Met genotype had higher mean levels of cortisol. More research is needed to examine the effect of COMT on CAR and the implications for health. Yet, due to the influence of COMT on CAR, it could be relevant to investigate whether patients experiencing goal disturbance have a specific COMT variant. These patients may react differently to goal disturbance and form a risk group for adverse cortisol levels. They should be monitored whether they may need extra care.

An additional finding was the significant relationship with the Met/Met COMT variant and DCS 7 months post-diagnosis. Closer inspection of the data showed that patients with the Met/Met variant had a more flattened DCS than patients with the Val/Val and Val/Met variants. This finding is in line with our hypothesis and supports the assumption that Met/Met carriers may be more insensitive to the endocrine reaction of the brain on stress and less able to degrade cortisol levels. Previous studies found that flattened DCS were linked to earlier mortality in cancer patients [23,26]. If indeed Met/Met carriers have more flattened DCS during the first months post-diagnosis, it could be that COMT variant is a risk factor in cancer patients. Although these suggestions are preliminary, this is a potentially interesting finding worth exploring in the future.

The present study has several strengths. First, we investigated the influence of goal disturbance on cortisol to gain information on the association with physiological functioning. Hereby, we complement research using self-reported health, making a step in understanding the relation between physiological and psychological processes in cancer patients. Also, we predicted two measures of cortisol (CAR and DCS), to assess the influence of goal disturbance on different diurnal cortisol responses. Further, we averaged CAR and DCS from two consecutive days, making it a more robust outcome measure. Lastly, we assessed cortisol longitudinally: 7 and 18 months post-diagnosis. Therefore we were able to investigate the long-term effect of goal disturbance on cortisol. Some limitations merit attention. First, the sample size was rather small, as we reduced the sample to eliminate possible contaminating sources to make the results as reliable as possible. While the sample remained comparable to the original sample, reducing the sample could have led to less power for the analyses. Second, due to the number of statistical tests, there could be a chance of an inflated Type I error. However, considering the novelty of the research topic, statistically correcting for multiple comparisons may be unnecessarily conservative. Third, we used the common method of calculating a difference score to assess CAR, without taking the awakening sample as a reference value as has sometimes been suggested (47). While both measures are possible, this may still have influenced the results. Lastly, the use of certain medications could have an influence on HPA-axis functioning [63], and smoking could have influenced cortisol levels [49]. We did not control for these factors and this could have affected the results.

We have found the first suggestions of the effect of goal disturbance in cancer patients on cortisol levels. Future research could further explore pathways with other biological indicators of well-being as well. Additionally, it could be interesting to investigate whether these findings are restricted to goal disturbance in cancer patients or can be found in patients with other (chronic) illnesses as well. The potential health risks of adverse cortisol levels due to goal disturbance highlights the need to study how cancer patients can adjust their goals and maintain better physical well-being. Additionally, as previous studies have shown that polymorphisms in other genes (e.g. DAT and MAO-A) also modulate stress responses [30,31], including these in the analysis could give further insight in the interaction between genes and stress responses [30,31].

In conclusion, the present study is the first to show that goal disturbance after cancer diagnosis may lead to increased cortisol awakening responses on the long term, but that this effect is less pronounced for patients with the Met/Met COMT genotype. Future studies are needed to further support these findings.

## Supporting Information

S1 Dataset (XLS)

S1 Text Manuscript under review with comparable data. (DOCX)
