# Supplementary material for: The Impact of Goal Disturbance after Cancer on Cortisol Levels over Time and the Moderating Role of COMT
Source: PLoS One. 2015 Aug 27;10(8):e0135708. doi: 10.1371/journal.pone.0135708 (PMC4552095; doi:10.1371/journal.pone.0135708)
Supplement: S1 Text — (DOCX) [file pone.0135708.s002.docx]

**Introduction**

Goals, their pursuit and achievement, are important as they give meaning and direction to people’s lives [1, 2]. Evidence from cross-sectional studies shows that the diagnosis of a severe illness such as cancer, can lead to disturbances in goal pursuit, and that such goal disturbance is related to poorer well-being [3-5]. Whether goal disturbance continues to impact well-being over time however, and what may moderate this impact, is still unknown. Theory assumes that when goal disturbance occurs, people need to adjust their goals to what is attainable to keep acceptable levels of well-being (e.g. [6]). Yet, whether goal adjustment moderates the relation between goal disturbance and well-being over time, has not been investigated. Hence, the current study will be the first to longitudinally investigate the predictive value of goal disturbance after cancer on well-being, and test the theoretical assumption of the moderating role of goal adjustment. What is more, in addition to using the conventional operationalisation of goal adjustment which assesses how people believe they will adjust their goals, the present study will also apply a novel approach assessing how people actually adjust their goals.

After a colorectal cancer diagnosis, physical problems could lead to difficulties in attaining goals and frequent hospital visits may leave less time in which goals can be pursued [5]. In general, goal disturbance was found to decline over time in people diagnosed with all stages of cancer [7, 8]. Yet there are indications that up to 18 months post-diagnosis, patients still report more health-related barriers to goal pursuit than healthy controls [8], thus possibly affecting well-being over that same period as well. Research is therefore needed investigating the long-term adverse effect of goal disturbance on well-being.

As most people with cancer deal with their illness rather well, adjustment of disturbed goals may play a role in maintaining well-being. To date, studies empirically investigating goal adjustment in people with cancer have focused almost exclusively on goal adjustment tendencies [4, 9-12]. Goal adjustment tendencies, or capacities, refer to the ease with which one believes to be able to disengage from disturbed goals and engage in new attainable ones, and is most commonly measured by the Goal Disengagement and Reengagement Scale (GDRS) [13]. The tendencies can be assessed in general or in reference to specific situations, such as adjustment to cancer. It was commonly found that goal re-engagement was related to better well-being, but goal disengagement was not [4, 9, 11, 12, 14]. We therefore hypothesise that higher dispositional re-engagement may help patients maintain well-being when facing goal disturbance due to cancer.

How people believe they will adjust, may not necessarily reflect how they actually adjust their goals. It is still unknown how goals really change or remain the same over time, and how this relates to well-being. Indeed, lately there have been repeated calls for long term studies of actual goal adjustment to extend and complement goal research (e.g. [12, 15, 16]).

Four theories could be said to form the basis of goal adjustment: *the dual-process model of assimilative and accommodative coping* (e.g. [17, 18]), *the model of selection, optimization, and compensation* (*SOC*, e.g. [19-21]), *the life-span theory of control* (e.g. [22-24]), and *control theory* (e.g. [1, 2]). These theories mention several specific strategies people may use when adjusting their goals. The use of these strategies can be determined by systematically investigating personal goals over time [25], as all strategies imply a change in, or stability of, a person’s goals. Investigating the use of goal adjustment strategies can therefore serve as a measure for actual goal adjustment. Six beneficial adjustment strategies were deducted from literature: *Shift priorities across life domains, Scale back goals in the same life domain, Scale up goals in the same life domain, Give up effort but remain committed / Put goals on hold*, *Form shorter term goals* and *Form longer term goals* [2, 6, 18, 24, 26, 27]. Their use is thought to be beneficial as they imply the continued engagement in important and attainable goals [6], but this has not yet been empirically examined.

Being capable of using a larger repertoire of adaptive goal adjustment strategies, instead of only one preferred strategy, has been suggested to benefit well-being [28, 29]. Flexibly deploying adjustment strategies enables people to respond to changing situations. Consequently, the more adjustment strategies are used after goals have been disturbed, the more this may help patients to maintain well-being. The flexible use of goal adjustment strategies will therefore be operationalised as the number of actual adjustment strategies used. The potentially beneficial role of goal adjustment within the relation between goal disturbance and well-being can thus be investigated. However, some strategies may be more beneficial than others. As the relations between the separate adjustment strategies and well-being is yet unknown, these will be examined as well.

Although higher dispositional re-engagement capacities and the use of more goal adjustment strategies are in general thought to be beneficial for well-being, the extent of this effect may depend on the specific situation in which they are required or used. It may be necessary to re-engage into new goals or use many adjustment strategies during the first chaotic months after diagnosis (i.e. the treatment period), as coming to terms with the initial diagnosis and consequences of the illness may require the quick adjustment of many goals (early loss-based selection) [8]. When facing (early) survivorship or end-of-life during the phase thereafter (i.e., the follow-up period) [30, 31], adopting new goals or adjusting goals may be somewhat less urgent, as adaptation to the most sudden life changes has already taken place. Higher goal re-engagement capacities and the use of more goal adjustment strategies may thus be more beneficial during the treatment period than the follow-up period.

In sum, the present study aims to answer the following research questions: 1) Does goal disturbance within a month post-diagnosis predict well-being 7 months post-diagnosis (i.e. the treatment period), and does goal disturbance 7 months post-diagnosis predict well-being 18 months post-diagnosis (i.e. the follow up period), and 2) Does goal adjustment (i.e. goal adjustment tendencies and number of beneficial actual goal adjustment strategies used) moderate the relation between goal disturbance and well-being over the treatment period and the follow-up period? With respect to these research questions, the following hypotheses were formulated: 1) Goal disturbance predicts well-being over both periods, with more goal disturbance leading to poorer well-being, and 2) A higher tendency to re-engage and the flexible use of more actual goal adjustment strategies will buffer the adverse effect of goal disturbance on well-being. It is assumed that this effect will be visible during both the treatment and follow-up periods, but will be more pronounced during the treatment period. Figure 1 depicts the research design guiding this study.

Insert Figure 1 here

**Methods**

***Design & Participants***

Between September 2011 and March 2013, all newly diagnosed people with medically confirmed colorectal cancer from four participating hospitals in the Netherlands were invited to participate. Exclusion criteria were: insufficient understanding of the Dutch language, drugs- and/or alcohol related problems, a cognitive impairment or psychiatric disorder, and being under the age of 18. There were three assessment points: within one month, 7 and 18 months post-diagnosis. The treatment period was defined as the period between one month and 7 months post-diagnosis, and the follow-up period between 7 and 18 months post-diagnosis. The study was approved by the medical ethical committee of a university medical centre in the Netherlands and all patients provided informed consent.

***Procedure***

A member of the hospital staff explained the study to eligible patients and handed them an information package. The package contained an information letter, an informed consent form, and a pre-paid envelope. Respondents were asked to read the information at home and return the informed consent form if they agreed to participate. After informed consent was received, the researchers assigned a trained interviewer to the respondent to conduct interviews at all three assessments at a place of the patients’ choice, mostly their homes.

***Measures***

*Demographic characteristics*

During the first assessment, within a month post-diagnosis, information was collected concerning respondents’ age and gender.

*Goal disturbance*

At all three assessments, respondents were asked to list three to ten personal goals, explained to them as projects they were currently working on, activities they were busy with or plans they wanted to achieve (based on e.g. [1, 8]). Goal disturbance was assessed with a single item, answered for each goal: To which degree does your illness hinder you in achieving your goal? Answers were given on a 10-point Likert scale ranging from 1 (not at all) to 10 (very) (based on e.g. [1, 8]). Mean goal disturbance scores from all goals at each assessment were calculated per respondent.

*Goal adjustment*

*Goal adjustment tendencies* To assess the ease with which someone believes to be able to disengage from unattainable goals and re-engage into new, meaningful goals, respondents completed the Goal Disengagement and Re-engagement scale (GDRS, [13]). This questionnaire consists of 10 items, 4 measuring disengagement and 6 measuring re-engagement, to be answered on a 5-point scale ranging from 1 ‘almost never true’ to 5 ‘almost always true’. Goal adjustment tendencies were assessed one and 7 months following diagnosis. Cronbach’s alpha was .66 for goal disengagement and .91 for goal re-engagement at one month post-diagnosis and .76 for goal disengagement and .91 for goal re-engagement 7 months post-diagnosis.

*Goal adjustment* *strategies* The use of six goal adjustment strategies (*Shift priorities across domains, Scale back goals in same life domain, Scale up goals in same life domain, Give up effort but remain committed/Put goals on hold*, *Form shorter term goals* and *Form longer term goals*) was calculated for the two periods studied (i.e. the treatment and follow-up periods) in another study (Janse et al., in press). Goal characteristics, i.e. goal content and goal structure, were used to calculate the strategies. *Goal content* was coded by researchers and comprised life domain (physical, psychological, social, achievement, leisure) and level of abstraction (1 = very abstract – 4 = very concrete). *Goal structure* was rated by respondents and comprised importance, attainability and effort (1 = not at all – 10 = very much) and temporal range (1 = within a week – 9 = more than 2 years) (based on e.g. [1, 8]). Scoring formulas were developed for each strategy using those characteristics over time important for defining their use. For instance, for the strategy *Scale back goals in the same life domain*, the characteristics life domain and level of abstraction were used, making it possible to determine whether goals within the same life domain decreased in level of abstraction over time (for the complete operationalisation of each of the strategies, see Janse et al., in press). For the purpose of the current study, the flexible use of actual goal adjustment strategies was assessed by the total number of goal adjustment strategies used for each period (i.e. 0 = no goal adjustment strategies used – 6 = 6 goal adjustment strategies used).

*Well-being*

*Quality of life* QoL was assessed using the Global health status/QoL subscale of the EORTC QLQ-C30 [32]. The items were: ‘How would you rate your overall health during the past week’ and ‘How would you rate your overall quality of life during the past week?’ Patients answered these items on a 7-point Likert-scale ranging from ‘very bad’ to ‘excellent’.

*Emotional functioning* Emotional functioning was assessed using the emotional functioning subscale of the EORTC QLQ-C30 [32]. This subscale consisted out of four items, answered on a scale from 1 = not at all - 4 = very much. An example item is: ‘Did you worry?’

Scores of both scales were linearly transformed to a scale from 0 – 100, with higher scores indicating better QoL or emotional functioning.

***Data analysis***

First, descriptive statistics and repeated-measures analyses with time as a within-subjects factor were performed to examine changes in mean levels of goal disturbance, goal adjustment, QoL and emotional functioning within one month, 7 and 18 months post-diagnosis. Second, due to the novelty of the developed method for assessing actual goal adjustment, correlations between the commonly used goal disengagement and re-engagement tendencies and the number of actual goal adjustment strategies were assessed. Then, four separate hierarchical regression analyses were performed. First, the predictive value of goal disturbance within one month post-diagnosis on QoL 7 months post-diagnosis was assessed, and the moderating role of goal adjustment tendencies at diagnosis and actual goal adjustment during the treatment period. Second, this same analysis was performed with emotional functioning as the outcome measure. Third, the predictive value of goal disturbance 7 months post-diagnosis on QoL 18 months post-diagnosis was assessed, and the moderating role of goal adjustment tendencies 7 months post-diagnosis and actual goal adjustment during the follow-up period. Finally, this same analysis was performed with emotional functioning as the outcome measure. Pearson correlations were used to check whether to control for socio-demographic variables. Age correlated significantly with goal disturbance, goal adjustment tendencies and both well-being measures and was entered in step one of the analyses. Then, goal disturbance (step 2), goal disengagement and re-engagement (step 3) and the use of goal adjustment strategies (step 4) were entered.

To investigate the possible interaction effects and to increase interpretability, the independent variable and potential moderators were centered, meaning that from each data-point, the mean was subtracted. These variables were then used to create new interaction variables, which were entered into step five of the regression analyses. Additionally, each goal adjustment strategy was also independently investigated as a moderator in separate analyses. Results were tested two-sided and a p-value of <.05 was considered significant throughout. Statistical Package for Social Sciences (IBM SPSS) version 22.0 for Windows was used for the statistical analysis.

**Results**

***Patients***

During the inclusion period, 622 eligible patients were identified. Three hundred eighty patients received information, of which 228 signed informed consent (response rate: 45.9%). Over time, 219 patients completed the first assessment, 201 completed the second assessment and 186 completed all three assessments (dropout rate of 15.1%). Of the 186 respondents, 39.2% were female, and the mean age was 64.2 years (for the complete flowchart, see Janse et al., in press).

***Well-being, goal disturbance, and goal adjustment over time***

Respondents reported a significantly improved QoL (*F* = 11.3, *p* < .001) and emotional functioning (*F* = 26.53, *p* < .001) over time. Additionally, they reported significantly less goal disturbance from 1 – 18 months post-diagnosis *(F* = 21.85, *p* < .001). Mean scores on the GDRS subscales remained stable and patients used more goal adjustment strategies during the follow-up period compared to the treatment period (*t* = -2.78, *p* = .01) (see Table 1).

Insert Table 1 here.

During the treatment period, 80.6% of respondents used a goal adjustment strategy, while during the follow-up period, 87. 6% used a strategy. Respondents who used a strategy during the treatment period, mostly used one strategy (44.6%). Twenty-six percent used two strategies, 7% used three strategies and 2.7% used four strategies. During the follow-up period, 38.2% used one strategy, 34.9% used two strategies, 11.3% used three strategies and 3.2% used four strategies.

***Goal adjustment measures***

Higher scores on goal disengagement within a month post-diagnosis was found to be significantly correlated with the use of less actual goal adjustment strategies during the treatment period (*r* = -.17, *p* = .02. Higher goal re-engagement scores within a month post-diagnosis were significantly correlated with the use of more goal adjustment strategies during the follow-up period (*r* =.16, *p* = .03).

***Does goal disturbance predict well-being over time?***

*Treatment period*

Hierarchical regression analyses for predicting well-being 7 months post-diagnosis (see Table 2) revealed the significant contribution of age in the prediction of QoL in the first, second and final step, explaining 5% all the total variance. Goal disturbance significantly predicted QoL in steps two to five, adding 7% of the total variance in QoL-scores, with more goal disturbance being associated with decreased QoL. With respect to emotional functioning, age significantly predicted higher emotional functioning in the first and second step, explaining 4% of the total variance. Higher goal disturbance significantly predicted lower emotional functioning in steps two to five, adding another 6% of the total variance.

Insert Table 2 here

*Follow-up period*

During the follow-up period (see Table 3), higher goal disturbance significantly predicted lower QoL in steps two to five, explaining 9% of the total variance. Older age significantly predicted higher emotional functioning in step one, explaining 2% of the total variance. Higher goal disturbance significantly predicted lower emotional functioning in steps two to five, explaining 4% of the total variance.

Insert Table 3 here

***Do goal adjustment tendencies and number of actual goal adjustment strategies used moderate the relation between goal disturbance and well-being?***

*Treatment period*

Three potential interactions (i.e. goal disturbance × goal disengagement, goal disturbance × goal re-engagement, goal disturbance × number of goal adjustment strategies), were entered to first predict Qol. Table 2 shows that only goal disengagement significantly moderated the relation between goal disturbance and QoL, indicating that higher scores on goal disengagement buffered the adverse effect of goal disturbance and leading to higher QoL. Investigating each goal adjustment strategy independently as a potential moderator, showed no significant results (data not shown).

With respect to emotional functioning, only the number of goal adjustment strategies used was a significant moderator (see Table 2). The negative effect of goal disturbance on emotional functioning was less for respondents who used more goal adjustment strategies. Investigating each goal adjustment strategy independently as a moderator, showed that the use of the strategy *Scale up goals in the same life domain* moderated the relation between goal disturbance and emotional functioning (b = 3.42, SE = 1.45, t = 2.36, *p* = .02), suggesting that the use of this strategy buffered the adverse effect of goal disturbance on emotional functioning (data not shown).

*Follow-up period*

None of the three potential interactions entered in step 5 significantly moderated the relation between goal disturbance and QoL, or goal disturbance and emotional functioning. The results of the analyses investigating adjustment strategies independently as a moderator, showed no significant results (data not shown).

**Discussion**

The present study set out to longitudinally investigate the theoretical assumptions that goal disturbance negatively impacts well-being, and that goal adjustment buffers this effect. The results show that, in line with our hypothesis, higher levels of goal disturbance indeed predicted lower levels of well-being between 1 – 7 months post-diagnosis (i.e. the treatment period), as well as between 7 – 18 months post-diagnosis (i.e. the follow-up period). Additionally, a higher tendency to disengage and the flexible use of more actual goal adjustment strategies, buffered the adverse effect of goal disturbance during the treatment period..

The findings of the current study are in line with previous cross-sectional studies demonstrating the adverse effect of goal disturbance on well-being [3-5]. Moreover, they show that goal disturbance is a consistent predictor of quality of life (QoL), as well as emotional functioning, up to 18 months after diagnosis. These results stress the importance of goal disturbance after cancer in determining well-being. However, it could be suggested that goal disturbance was not particularly high at any assessment point (i.e. the maximum mean score was 4.7 (SD 2.4) on a scale from 1 to 10 at the first assessment). As first assessment point was within one month after cancer diagnosis, patients could have already started adjusting their goals in the time between diagnosis and the first assessment, in line with the model of immediate loss-based selection [8, 19]. Also, according to theories of life-span development, higher age is related to decreasing opportunities for goal achievement. People can anticipate this by adjusting their goals to match decreasing resources [24]. As the older patients in our sample may already started adjusting their goals, they could have experienced lower levels of goal disturbance.

With respect to the moderating effect of goal adjustment tendencies, results showed that higher dispositional goal disengagement, and not goal re-engagement, was beneficial for QoL during the treatment period. This is in contrast to prior, mostly cross-sectional, research in people with cancer, that found beneficial effects only for goal re-engagement and not for goal disengagement [4, 11, 12, 14]. Yet, these studies focused on cancer survivors who were assessed at various times since diagnosis, i.e. from ten months [14] to seven years [11, 12]. It may be that goal disengagement is particularly adaptive in the treatment period during the first months following cancer diagnosis. During these hectic months, patients may need to (temporarily) let go of their previously important goals to be able to focus on treatment and coming to terms with their cancer diagnosis. Still, the effects of goal disengagement were modest and future research is needed to confirm these findings.

Flexibility in using actual goal adjustment strategies during the treatment period led to better emotional functioning when goal disturbance was high. Other studies already found beneficial effects of several measures of coping flexibility [28, 29], and the current findings of actual goal adjustment build on these results. We found significant results only during the treatment period. During the first months of diagnosis and treatment, more choices and considerations might be necessary to deal with goal disturbance. While it is in general thought that people adjust their goals throughout their life, this may be extra important following a cancer diagnosis. During the year thereafter, it may be less urgent to react to sudden goal disturbance, but more to permanently changed life circumstances. Goal adjustment may then again be part of natural and developmental adjustment, and has less added value.

The current study has several strengths, namely a large sample size, longitudinal design and novel approach towards assessing actual goal adjustment. The validity of the method to investigate the use of the actual goal adjustment strategies is not established, and this could be seen as a limitation even though the method was tested in an earlier study [25].

Findings of the present study provide directions for future research. As the effect of goal disturbance and adjustment on the two different well-being measures (i.e. QoL and emotional functioning) differed, more research is needed to investigate the mechanisms behind the different goal adjustment measures and how they relate to well-being measures. In addition, as touched upon earlier, age could cause differences in goal disturbance and adjustment. It could thus be relevant to investigate goal disturbance and adjustment, as well as their impact on well-being, in younger patient samples. Furthermore, as the current study found support for a long-term adverse effect of goal disturbance, it seems especially important to continue to study how goal adjustment may help patients maintain well-being. Considering that this study has made only the first steps towards testing existing theories on goal disturbance and adjustment, and the explained variance in our models remained modest, future research is needed to replicate and extend these findings.

With respect to the clinical implications, we found indications that both higher reported general goal disengagement capacities and the use of more actual goal adjustment strategies could be beneficial after goal disturbance. Goal disengagement however, assesses a general and stable trait, and might therefore be difficult to intervene upon. On the other hand, the novel method of adjustment strategies provides clear suggestions of concrete actions which can be practiced in psychological interventions. Also, when offering interventions focusing on goal adjustment, the current results suggest that it seems to be important to do so within the first months following diagnosis. The study therefore adds new pieces of knowledge on what may be beneficial for patients’ well-being at specific time points after a cancer diagnosis.

In sum, the present study has made a step in advancing the field of goal research by answering to the call for longitudinal studies on goal disturbance, actual goal adjustment and well-being. Findings highlight the relevance of focusing on what actually happens to goals after goal disturbance due to cancer in specific periods after diagnosis, and how this influences well-being.

**References**

1. Emmons, R. A. (1999). The psychology of ultimate concerns: Motivation and spirituality in personality. New York, NY US: Guilford Press.

2. Carver, C. S., & Scheier, M. F. (1998). On the self-regulation of behavior. New York, NY US: Cambridge University Press.

3. Muller, L., & Spitz, E. (2010). Effects of personal goal disturbance on psychological distress. European Review of Applied Psychology/Revue Européenne de Psychologie Appliquée, 60(2), 105-112.

4. Offerman, M. P. J., Schroevers, M. J., van der Velden, L., de Boer, M. F., & Pruyn, J. F. A. (2010). Goal processes & self-efficacy related to psychological distress in head & neck cancer patients and their partners. European Journal of Oncology Nursing, 14(3), 231-237.

5. Stefanic, N., Caputi , P., & Iverson, D. C. (2014). Investigating physical symptom burden and personal goal interference in early-stage breast cancer patients. Supportive Care In Cancer: Official Journal Of The Multinational Association Of Supportive Care In Cancer, 22(3).

6. Haase, C. M., Heckhausen, J., & Wrosch, C. (2013). Developmental regulation across the life span: Toward a new synthesis. Developmental psychology, 49(5), 964-972.

7. Janse, M., Ranchor, A. V., Smink, A., Sprangers, M. A., & Fleer, J. (2015). Changes in cancer patients' personal goals in the first 6 months after diagnosis: the role of illness variables. Supportive care in cancer: official journal of the Multinational Association of Supportive Care in Cancer, 23(7), 1893-1900.

8. Pinquart, M., Fröhlich, C., & Silbereisen, R. K. (2008). Testing models of change in life goals after a cancer diagnosis. Journal of Loss and Trauma, 13(4), 330-351.

9. Zhu, L., Ranchor, A. V., Van der Lee, M., Garssen, B., Sanderman, R., & Schroevers, M. J. (2015). The role of goal adjustment in symptoms of depression, anxiety and fatigue in cancer patients receiving psychosocial care: a longitudinal study. Psychology & Health, 30(3).

10. Schroevers, M. J., Kraaij, V., & Garnefski, N. (2011). Cancer patients' experience of positive and negative changes due to the illness: relationships with psychological well-being, coping, and goal reengagement. Psycho-oncology, 20(2), 165-172.

11. Schroevers, M., Kraaij, V., & Garnefski, N. (2008). How do cancer patients manage unattainable personal goals and regulate their emotions?. British Journal of Health Psychology, 13(3), 551-562.

12. Thompson, E., Stanton, A. L., & Bower, J. E. (2013). Situational and dispositional goal adjustment in the context of metastatic cancer. Journal of personality, 81(5), 441-451.

13. Wrosch, C., Scheier, M. F., Miller, G. E., Schulz, R., & Carver, C. S. (2003). Adaptive Self-Regulation of Unattainable Goals: Goal Disengagement, Goal Reengagement, and Subjective Well-Being. Personality and Social Psychology Bulletin, 29(12), 1494-1508.

14. Wrosch, C., & Sabiston, C. M. (2013). Goal adjustment, physical and sedentary activity, and well-being and health among breast cancer survivors. Psycho-oncology, 22(3), 581-589.

15. Von Blanckenburg, P., Seifart, U., Conrad, N., Exner, C., Rief, W., & Nestoriuc, Y. (2014). Quality of life in cancer rehabilitation: the role of life goal adjustment. Psycho-oncology.

16. Wrosch, C., Amir, E., & Miller, G. E. (2011). Goal adjustment capacities, coping, and subjective well-being: The sample case of caregiving for a family member with mental illness. Journal of personality and social psychology.

17. Brandtstädter, J., & Renner, G. (1990). Tenacious goal pursuit and flexible goal adjustment: Explication and age-related analysis of assimilative and accommodative strategies of coping. Psychology and aging, 5(1), 58-67.

18. Brandtstädter, J., & Rothermund, K. (2002). The life-course dynamics of goal pursuit and goal adjustment: A two-process framework. Developmental Review, 22(1), 117-150.

19. Baltes, P. B., & Baltes, M. M. (1990). Psychological perspectives on successful aging: The model of selective optimization with compensation. In P. B. Baltes, M. M. Baltes, P. B. Baltes, & M. M. Baltes (Eds.), Successful aging: Perspectives from the behavioral sciences. (pp. 1-34). New York, NY US: Cambridge University Press.

20. Freund, A. M. (2008). Successful aging as management of resources: The role of selection, optimization, and compensation. Research in Human Development, 5(2), 94-106.

21. Freund, A. M., & Baltes, P. B. (2002). Life-management strategies of selection, optimization and compensation: Measurement by self-report and construct validity. Journal of personality and social psychology, 82(4), 642-662.

22. Heckhausen, J., & Schulz, R. (1995). A life-span theory of control. Psychological review, 102(2), 284-304.

23. Schulz, R., & Heckhausen, J. (1996). A life span model of successful aging. American Psychologist, 51(7), 702-714.

24. Heckhausen, J., Wrosch, C., & Schulz, R. (2010). A motivational theory of life-span development. Psychological review, 117(1), 32-60.

25. Janse, M., Sulkers, E., Tissing, W. J. E., Sanderman, R., Sprangers, M. A. G., Ranchor, A. V., et al. (2014). Goal adjustment strategies operationalised and empirically examined in adolescents with cancer. Journal of Health Psychology, E-pub ahead of print.

26. Boerner, K., & Jopp, D. (2007). Improvement/maintenance and reorientation as central features of coping with major life change and loss: Contributions of three life-span theories. Human development, 50(4), 171-195.

27. Wrosch, C., Scheier, M. F., Carver, C. S., & Schulz, R. (2003). The importance of goal disengagement in adaptive self-regulation: When giving up is beneficial. Self and Identity, 2(1), 1-20.

28. Arends, R. Y., Bode, C., Taal, E., & Van, d. L. (2013). The role of goal management for successful adaptation to arthritis. Patient education and counseling, 93(1), 130-138.

29. Cheng, C., Lau, H. B., & Chan, M. S. (2014). Coping flexibility and psychological adjustment to stressful life changes: A meta-analytic review. Psychological bulletin, 140(6), 1582-1607.

30. Henselmans, I., Helgeson, V. S., Seltman, H., de Vries, J., Sanderman, R., & Ranchor, A. V. (2010). Identification and prediction of distress trajectories in the first year after a breast cancer diagnosis. Health Psychology, 29(2), 160-168.

31. Stanton, A. L., Ganz, P. A., Rowland, J. H., Meyerowitz, B. E., Krupnick, J. L., & Sears, S. R. (2005). Promoting adjustment after treatment for cancer. Cancer, 104(11 (Suppl)), 2608.

32. Aaronson, N. K., Ahmedzai, S., Bergman, B., Bullinger, M., Cull, A., Duez, N. J., et al. (1993). The European Organisation for Research and Treatment of Cancer QLQ-C30: A quality-of-life instrument for use in international clinical trials in oncology. Journal of the National Cancer Institute, 85, 365-376.

**Tables**

**Table 1.** Data for QoL, emotional functioning, goal disturbance and adjustment over time (n=186)

| Variable | M (SD) Time 1:  Within 1 month post- diagnosis | M (SD) Time 2:  6 months later | M (SD) Time 3:  18 months later | | F (*p*)^[[1]](#footnote-1)^ |
| --- | --- | --- | --- | --- | --- |
| Quality of life (EORTC) | 72.8 (20.8) | 76.6 (19.1) | 80.1 (17.9) | **11.3 (<.001)** | |
| Emotional functioning (EORTC) | 75.6 (20.1) | 83.1 (19.2) | 85.1 (19.2) | **26.53 (<.001)** | |
| Goal disturbance | 4.7 (2.4) | 4.1 (2.7) | 3.3 (2.6) | **21.85 (<.001)** | |
| Goal adjustment tendencies |  |  |  | **t (*p*)^[[2]](#footnote-2)^** | |
| *Goal disengagement* | 11.9 (3.1) | 11.9 (3.1) |  | -.20 (.84) | |
| *Goal re-engagement* | 21.2 (4.6) | 21.3 (4.3) |  | -.42 (.68) | |
|  | **Period 1 (T1 – T2)** | **Period 2 (T2 – T3)** |  | **t (*p*)** | |
| N^o^ of Goal adjustment strategies | 1.3 (0.9) | 1.6 (1.0) |  | **-2.78 (.01)** | |

**Table 2.** Hierarchical regression analyses predicting well-being 7 months post-diagnosis (Time 2) controlling for age (step 1), and entering goal disturbance (step 2), goal disengagement and goal reengagement (step 3) and N^o^ of goal adjustment strategies (step 4) within a month post-diagnosis (Time 1). Interaction terms were entered in step 5.

|  | **Step 1** | | **Step 2** | | **Step 3** | | **Step 4** | | **Step 5** | |
| --- | --- | --- | --- | --- | --- | --- | --- | --- | --- | --- |
|  | **B (SE)** | **Beta** | **B (SE)** | **Beta** | **B (Se)** | **Beta** | **B (Se)** | **Beta** | **B (Se)** | **Beta** |
| **Quality of life Time 2** |  |  |  |  |  |  |  | |  |  |
| Age | .40 (.13) | .23**^[[3]](#footnote-3)^ | .28 (.13) | .16* | .27 (.14) | .15 | .27 (.14) | .15 | .30 (14) | .17* |
| Goal disturbance Time 1 | - |  | -2.16 (.58) | -.27** | -2.06 (.58) | -.26** | -2.06 (.58) | -.26** | -2.22 (.59) | -.28** |
| Goal disengagement Time 1 | - |  | - |  | .48 (.48) | .08 | .53 (.49) | .09 | .51 (.49) | .08 |
| Goal re-engagement Time 1 | - |  | - |  | .16 (.33) | .04 | .15 (.33) | .04 | .13 (.33) | .03 |
| N^o^ of Goal adjustment strategies Time 1- Time 2 | - |  | - |  | - |  | 1.02 (1.45) | .05 | 1.3 (1.5) | .06 |
| Goal disturbance × goal disengagement | - |  | - |  | - |  | - |  | .44 (.22) | .16* |
| Goal disturbance × goal re-engagement | - |  | - |  | - |  | - |  | -.17 (.12) | -.10 |
| Goal disturbance × N^o^ of goal adjustment strategies | - |  | - |  | - |  | - |  | .67 (.62) | .08 |
| ΔR^2^^[[4]](#footnote-4)^ | .05** |  | .07** |  | .01 |  | .00 | | .03 total = .16 | |
| **Emotional functioning Time 2** |  |  |  |  |  |  |  | |  | |
| Age | .37 (.13) | .21** | .26 (.13) | .15* | .22 (.14) | .12 | .22 (.14) | .12 | .25 (.14) | .15 |
| Goal disturbance Time 1 |  |  | -1.91 (.6) | -.24** | -1.78 (.58) | -.23** | -1.78 (.58) | -.23** | -1.82 (.58) | -.23** |
| Goal disengagement Time 1 |  |  |  |  | .97 (.48) | .16* | .9 (.49) | .15 | .9 (.49) | .15 |
| Goal reengagement Time 1 |  |  |  |  | .07 (.32) | .02 | .08 (.33) | .02 | .04 (.32) | .01 |
| N^o^ of Goal adjustment strategies Time 1-Time 2 |  |  |  |  |  |  | -1.22 (1.44) | -.06 | -1.01 (1.4) | -.05 |
| Goal disturbance × goal disengagement |  |  |  |  |  |  |  |  | .35 (.22) | .13 |
| Goal disturbance × goal re-engagement |  |  |  |  |  |  |  |  | -.08 (.12) | -.05 |
| Goal disturbance × N^o^ of goal adjustment strategies |  |  |  |  |  |  |  |  | 1.26 (.61) | .15* |
| ΔR^2^ | .04** |  | .06** |  | .03 |  | .00 | | .03 total = .16 | |

**Table 3.** Hierarchical regression analyses predicting well-being 18 months post-diagnosis (Time 1) controlling for age (step 1), and entering goal disturbance (step 2), goal disengagement and goal reengagement (step 3) and N^o^ of goal adjustment strategies (step 4) 7 months post-diagnosis (Time 1). Interaction terms were entered in step 5.

|  | **Step 1** | | **Step 2** | | **Step 3** | | **Step 4** | | **Step 5** | |
| --- | --- | --- | --- | --- | --- | --- | --- | --- | --- | --- |
|  | **B (SE)** | **Beta** | **B (SE)** | **Beta** | **B (Se)** | **Beta** | **B (Se)** | **Beta** | **B (Se)** | **Beta** |
| **Quality of life Time 3** |  |  |  |  |  |  |  | |  |  |
| Age | -.03 (.12) | -.02 | -.11 (.12) | -.07 | -.09 (.13) | -.06 | -.13 (.13) | -.08 | -.12 (.13) | -.07 |
| Goal disturbance Time 2 | - |  | -2.02 (.49) | -.3**^[[5]](#footnote-5)^ | -1.87 (.5) | -.28** | -1.99 (.49) | -.29** | -1.96 (.5) | -.29** |
| Goal disengagement Time 2 | - |  | - |  | .25 (.45) | .04 | .37 (.45) | .06 | .32 (.46) | .05 |
| Goal re-engagement Time 2 | - |  | - |  | .45 (.33) | .11 | .34 (.33) | .08 | .34 (.33) | .08 |
| N^o^ of Goal adjustment strategies Time 2- Time 3 | - |  | - |  | - |  | 3.01 (1.34) | .16* | 2.96 (1.34) | .16* |
| Goal disturbance × goal disengagement | - |  | - |  | - |  | - |  | -.02 (.16) | -.01 |
| Goal disturbance × goal re-engagement | - |  | - |  | - |  | - |  | .12 (.11) | .08 |
| Goal disturbance × N^o^ of goal adjustment strategies | - |  | - |  | - |  | - |  | -.17 (.52) | -.02 |
| ΔR^2^^[[6]](#footnote-6)^ | .00 |  | .09** |  | .02 |  | .03* | | .01 total = .15 | |
| **Emotional functioning Time 3** |  |  |  |  |  |  |  | |  | |
| Age | .26 (.13) | .15* | .2 (.13) | .11 | .16 (.14) | .09 | .13 (.14) | .08 | .15 (.14) | .09 |
| Goal disturbance Time 2 | - |  | -1.46 (.53) | -.2** | -1.24 (.53) | -.17* | -1.34 (.53) | -.19* | -1.23 (.54) | -.18* |
| Goal disengagement Time 2 | - |  | - |  | .87 (.49) | .14 | .97 (.49) | .16 | .87 (.50) | .14 |
| Goal reengagement Time 2 | - |  | - |  | .18 (.35) | .04 | .1 (.36) | .02 | .07 (.36) | .02 |
| N^o^ of Goal adjustment strategies Time 2-Time 3 | - |  | - |  | - |  | 2.36 (1.47) | .12 | 2.17 (1.48) | .11 |
| Goal disturbance × goal disengagement | - |  | - |  | - |  | - |  | -.07 (.18) | -.03 |
| Goal disturbance × goal re-engagement | - |  | - |  | - |  | - |  | .22 (.12) | .14 |
| Goal disturbance × N^o^ of goal adjustment strategies | - |  | - |  | - |  | - |  | -.23 (.56) | -.03 |
| ΔR^2^ | .02* |  | .04** |  | .02 |  | .01 | | .02 total = .11 | |

1. Repeated measures GLM with 3 factors, factor = time [↑](#footnote-ref-1)
2. Paired sample t-test [↑](#footnote-ref-2)
3. *p<0.05, **p<0.01 [↑](#footnote-ref-3)
4. Percentage of variance explained by the model [↑](#footnote-ref-4)
5. *p<0.05, **p<0.01 [↑](#footnote-ref-5)
6. Percentage of variance explained by the model [↑](#footnote-ref-6)
